# Harnessing *Pelargonium Odoratissimum* Extracts for Sustainable Ground Meat Preservation

**DOI:** 10.1111/1750-3841.70597

**Published:** 2025-10-15

**Authors:** Anis Ben Hsouna, Boutheina Ben Akacha, Monika Michalak, Narjes Baazaoui, Wirginia Kukula‐Koch, Wojciech Koch, Rania Ben Saad, Miroslava Kačániová, Stefania Garzoli

**Affiliations:** ^1^ Laboratory of Biotechnology and Plant Improvement Centre of Biotechnology of Sfax Sfax Tunisia; ^2^ Department of Environmental Sciences and Nutrition, Higher Institute of Applied Sciences and Technology of Mahdia University of Monastir Monastir Tunisia; ^3^ Department of Pharmaceutical Science, Collegium Medicum Jan Kochanowski University Kielce Poland; ^4^ Biology Department, Faculty of Science King Khalid University Abha Saudi Arabia; ^5^ Tissue Culture and Cancer Biology Research Laboratory King Khalid University Abha Saudi Arabia; ^6^ Department of Pharmacognosy with Medicinal Plants Garden Medical University of Lublin Lublin Poland; ^7^ Department of Food and Nutrition Medical University of Lublin Lublin Poland; ^8^ Institute of Horticulture, Faculty of Horticulture and Landscape Engineering Slovak University of Agriculture Nitra Slovakia; ^9^ School of Medical & Health Sciences VIZJA University Warszawa Poland; ^10^ Department of Chemistry and Technologies of Drug Sapienza University Rome Italy

**Keywords:** bioactive compounds, ethyl acetate, natural preservatives, phenolic compounds, sustainable preservation

## Abstract

*Pelargonium odoratissimum* is traditionally recognized for its medicinal properties, yet its potential in food preservation remains underexplored. This study evaluated the phytochemical composition, antioxidant capacity, and antibacterial activity of its solvent extracts, with emphasis on the ethyl acetate fraction. The extract was rich in bioactive compounds, containing 11.84 µg GAE/g of total polyphenols and 3.08 µg QE/g of total flavonoids. Antibacterial testing demonstrated strong inhibitory effects, with minimum inhibitory concentrations ranging from 2.5 to 6.25 mg/mL and minimum bactericidal concentrations between 3.12 and 7.5 mg/mL, confirming bactericidal potential. Antioxidant assessment using the DPPH assay revealed potent radical‐scavenging activity (IC50 = 10.65 µg/mL). When incorporated as a natural preservative in ground beef, the ethyl acetate fraction effectively reduced microbial growth and lipid oxidation during 14 days of refrigerated storage (4°C), lowering aerobic plate counts by up to 2.74 log CFU/g compared with the control. Sensory evaluation further confirmed its ability to preserve meat color, odor, and texture. Collectively, these findings highlight *Pelargonium odoratissimum* ethyl acetate extract as a promising natural preservative for improving food quality, safety, and shelf life, offering a sustainable alternative to synthetic additives.

## 1 Introduction

The medicinal properties of *Pelargonium* species cannot easily be disregarded due to their remarkable healing capacity. *Pelargonium* species have been mentioned in many folklore documents, and numerous bioactive compounds have been identified (Ben Akacha et al. 2025; Ben Hsouna, Ben Akacha, et al. 2024). These interesting and hardy bushy plants offer their medicinal properties and can be used in preservation processes. Researching the healing capacities and properties of *Pelargonium odoratissimum* is of utmost importance, as the plant lists a wide range of medicinal uses, and this species is used alone, collectively, or in combination with other medicinal plants to treat a wide range of ailments (Ben Hsouna et al. 2024). Aligned with the rich folklore tradition, the knowledge of its antimicrobial potential can also be implemented in the preservation of foodstuffs (Kolodziej 2007). The tall, erect herbaceous *P. odoratissimum* plant is also widely known as the apple‐scented geranium due to its aromatic, scented leaves that smell like myrrh and apples when damaged (Akacha et al. 2025; Boukhatem et al. s. d.). *P. odoratissimum* plants grow less than 1 meter tall and have cream, green, hairy leaves with dark brown glandular spots across the leaf surface. The attractive pink flowers have brown spots and an oval five‐petaled arrangement, with no spurs, like the consistent flower structures of other *Pelargonium* species. The petals are rough and glitter when viewed up close. Pelargoniums are said to have originated from Africa, and this enchanting species with attractive scented leaves hails from the Eastern Cape (Ben Hsouna et al. 2024; Liang et al. 2023).

Thanks to their potential use in traditional medicine, several studies have evaluated plants in the *Pelargonium* genus for their biological effects in humans. *P. odoratissimum* is a lesser‐known species of the genus with its application in traditional medicine to treat respiratory problems. The tonic, refrigerant, vermifuge, astringent, antiseptic, diuretic, antispasmodic, relaxant, and stimulant actions had been attributed to *P. odoratissimum* so far. The roots, and leaves, as well as leaf and root infusion, and leaf decoction have been used in the treatment of stomach disorders, chest disorders, diarrhea, dysentery, sore throat, coughs, colds, kidney problems, menstrual cycle adjustment, and sexually transmitted diseases in various indigenous cultures (Abdelbaky et al. 2022; Lis‐Balchin 2002).

As it belongs to the *Pelargonium* genus known for its essential oil's antimicrobial activity (Andrade et al. 2011; T. Li et al. 2024; Shang et al. 2024), *P. odoratissimum* was proven to show antioxidant and antibacterial properties as well. Plant‐derived essential oils possess strong antimicrobial action and may serve as effective ingredients for food preservation (Ben Hsouna et al. 2024). Additionally, its antioxidant activity may help prevent lipid oxidation (Andrade et al. 2011).

Globally, meat remains a cornerstone of human nutrition, providing high‐quality protein, iron, vitamin B_12_, and other essential nutrients (Ben Akacha, Garzoli, et al. 2023; Ben Akacha et al. 2023). As of 2020, red meat (including beef) accounted for about 21 % of global meat consumption, with beef following poultry and pork (Siroli et al. 2020). In 2024, beef consumption reached roughly 75 million metric tons, making it the second largest meat type by volume after pork. Per capita beef consumption varies widely by region: Argentina leads with approximately 46–55 kg per person per year, followed by the United States (∼38 kg) and Brazil (∼35–37 kg). Globally, however, per capita beef consumption remains much lower, averaging around 6 kg annually in developing regions, compared to about 43 kg in North America and 34.6 kg in South America (Bifaretti et al. 2023; Kang et al. 2018).

Ground beef, while not always isolated in global statistic, bears the same vulnerabilities as beef in general: high surface‐area and lipid content accelerate microbial and oxidative degradation. Historically, synthetic antioxidants like BHA and BHT were used to prolong shelf life, but concerns over their health effects have prompted a shift toward biological preservatives (Ben Akacha et al. 2024; Ben Hsouna, Ben Akacha, et al. 2024; Bouteraa et al. 2023). Natural alternatives such as plant extracts (rosemary, grape seed) or antimicrobial metabolites from lactic acid bacteria offer promising, safer, and more sustainable solutions aligned with consumer preferences for cleaner labels and improved food safety (Moreno et Arteaga‐Miñano 2018; Zhang et al. 2024).

In light of these preliminary results, the biological potential of *P. odoratissimum* is certainly worth further research into the potential of other parts of the plant besides the flowers, the identification and purification of active ingredients, and the standardization extracts for the presence of the active compounds. *P odoratissimum* antimicrobial activity is linked to the presence of its essential oil. Further experiments are needed to verify the properties and to register other potential applications of the plant. This study aimed to evaluate the antimicrobial and antioxidant properties of *P. odoratissimum* extracts, particularly the ethyl acetate fraction, and assess their potential application as natural bio‐preservatives in food preservation. By determining the phytochemical composition, antibacterial efficacy, and antioxidant capacity of the extracts, this study seeks to explore their effectiveness in inhibiting microbial growth and lipid oxidation in meat products, thus providing a sustainable alternative to synthetic preservatives.

## 2 Materials and Methods

### 2.1 Collection and Preparation of Plant Extracts

*P. odoratissimum*, used in the present study, was collected in Tuscany (Italy, harvested in July 2022) and was provided by “Essenziale” Azienda Agricola, San Donato in Poggio (FI), Italy. The harvesting process involved a careful selection of the aerial parts, including the leaves and stems, to ensure optimal quality for subsequent analysis. After collection, the plant material was subjected to a drying process. The aerial parts were dried in a dark environment to preserve the bioactive compounds and avoid degradation due to light exposure. The drying was carried out at room temperature to prevent the loss of volatile compounds sensitive to high temperatures (Ben Hsouna et al., 2022). This method was chosen to maintain the integrity of the plant's chemical profile for further investigation

The bioactive compounds from *P. odoratissimum* were extracted using successive maceration with organic solvents of increasing polarity. A total of 1000 g of finely powdered aerial parts were subjected to sequential extraction with hexane (HEPO), dichloromethane (DEPO), and ethyl acetate (EAPO). For each extraction, the plant material was macerated with the solvent at a ratio of 1:10 (*w/v*), at room temperature conditions, for 48 h with continuous agitation to maximize compound solubility. After each step, the mixtures were filtered through Whatman No. 1 filter paper, and the solvents were evaporated under reduced pressure using a rotary evaporator at temperatures not exceeding 40°C to avoid degradation of thermolabile constituents (Palaiogiannis et al. 2023).

The extracts were then dried and weighed to calculate the respective extraction yields following the equation, indicating the efficiency of each solvent in extracting the bioactive components from the plant material (B. Akacha et al. 2022):

The extraction yield (%) = mass of the extract after freeze‐drying / mass of the dried plant material × 100.

The residue of each extract was kept at −20°C in the dark.

### 2.2 Phytochemical Analysis

#### 2.2.1 GC‐MS Analysis of *P. odoratissimum* Extracts

To describe the chemical composition of *P. odoratissimum* extracts, gas chromatograph equipped with FID (flame ionization detector) and coupled with a single quadrupole mass spectrometer (Clarus 500 model Perkin Elmer—Waltham, MA, USA) was used to carry out the analyses. A capillary column (varian factor four VF‐5) was housed in the GC oven whose programmed temperature was set initially at 70°C then a gradient of 6°C/min to 170°C, then a gradient of 8°C/min to 250°C for 15 min. The injector GC was set at 280°C. Helium was used as a carrier gas at a constant rate of 1 mL/min. MS detection was performed with electron ionisation (EI) at 70 eV operating in the full‐scan acquisition mode in the *m/z* range of 45–600 amu. The MS‐fragmentation pattern was compared to the NIST11 mass spectra library database to identify the volatile compounds. Further, the linear retention indices (LRIs) were calculated using a mixture of n‐alkanes (C_8_–C_30_ aliphatic hydrocarbons), injected under the same operating conditions. The relative amounts of the components were expressed as percent peak area relative to total peak area without using an internal standard and any factor correction. The analysis was carried out in triplicate.

#### 2.2.2 Determination of the Total Phenolic Content

The total polyphenol content of the EAPO extracts was determined spectrophotometrically using a modified Folin‐Ciocalteu colorimetric method [8,11]. To ensure measurements fell within the standard curve range (0.0–600.0 µg gallic acid/mL) (Takara, USA, Qiagen) all extracts were diluted 1:5 (*v/v*). For the assay, 125 µL of the standard gallic acid solution or the diluted EAPO extract (1:5 *v/v*) was mixed with 0.5 mL of distilled water in a test tube. Then, 125 µL of Folin‐Ciocalteu reagent was added, and the mixture was thoroughly vortexed. After standing for 6 minutes at room temperature, 1.25 mL of a 7% aqueous sodium carbonate solution was added. The volume was then adjusted to 3 mL with distilled water. The samples were incubated at room temperature for 90 minutes before absorbance was measured at 760 nm against a blank using a DYNEX MRX II spectrophotometer (DYNEX Technologies, Inc., Chantilly, USA). Gallic acid standards prepared under the same conditions were used to generate the calibration curve. Results were expressed as the mean ± standard deviation (micrograms of gallic acid equivalents per gram of EAPO) for three replicates.

#### 2.2.3 Determination of the Total Flavonoid Content

The total flavonoid content of the EAPO extract was measured using a colorimetric method. In summary, 0.25 mL of the EAPO extract or the standard (+)‐catechin solution was combined with 1.25 mL of distilled water in a test tube. Then, 75 µL of a 5% sodium nitrite (NaNO_2_) (Takara, USA, Qiagen) solution was added. After 6 minutes, 150 µL of a 10% aluminum chloride hexahydrate (AlCl_3_·6H_2_O) solution was introduced, and the mixture was left to react for an additional 5 min. Subsequently, 0.5 mL of 1 M sodium hydroxide (NaOH) was added. The final volume was adjusted to 2.5 mL with distilled water, and the solution was thoroughly mixed. The absorbance was recorded immediately at 510 nm against a blank using a DYNEX MRX II spectrophotometer (DYNEX Technologies, Inc., Chantilly, USA) (Khorasani Esmaeili et al. 2015). The flavonoid content was quantified by comparing the sample absorbance with a standard curve prepared using known concentrations of (+)‐catechin. Based on eight replicates, the results were expressed as the mean ± standard deviation (micrograms of catechin equivalents per gram).

### 2.3 Antioxidant Activity

#### 2.3.1 DPPH Radical Scavenging Activity

The antioxidant capacity of the extract was evaluated using the DPPH (2,2‐diphenyl‐1‐picrylhydrazyl) photocolorimetric assay. DPPH was purchased from Sigma‐Aldrich (St. Louis, MO, USA) with a purity of ≥95%.

This assay is based on the ability of antioxidants to scavenge the stable DPPH free radical, which exhibits a deep violet color that fades to yellow upon reduction. A DPPH stock solution (0.3 mM in ethanol) was freshly prepared daily and mixed with the extract at various final concentrations (250, 500, 750, and 1000 µg/mL). The mixtures were incubated in the dark at room temperature for 30 min. The decrease in absorbance was measured at 517 nm using a microplate reader, with a DPPH solution without extract serving as the negative control. Ascorbic acid (AA) was used as a positive control.

The radical scavenging activity was expressed as the percentage of DPPH inhibition, calculated using the following formula:

$$\%\;\mathrm{Inhibitions}=\left(A_{\mathrm{DPPH}}-A_{\mathrm{Extract}\;\mathrm{with}\;\mathrm{DPPH}}\right)/A_{\mathrm{DPPH}})\times \;100$$

where *A* is the absorbance.

The inhibitory concentration at 50% IC_50_ was calculated by linear regression.

### 2.4 Antibacterial Testing Assays

#### 2.4.1 Bacterial Strains and Culture Conditions

Authentic pure bacterial cultures were obtained from international culture collections: the American Type Culture Collection (ATCC) and the local collection of the Biotechnology Center of Sfax, Tunisia. They included Gram‐positive and Gram‐negative bacteria, *Bacillus cereus* ATCC 14579, *Staphylococcus aureus* ATCC 25923, *Enterococcus faecalis* ATCC 29212, *Micrococcus luteus* ATCC 1880, *Listeria monocytogenes* ATCC 19117, *Salmonella enterica* ATCC 43972, *Escherichia coli* ATCC 25922, and *Pseudomonas aeruginosa* ATCC 9027. The bacteria were cultured on MH agar at 37°C for 12 to 24 h, except for *Bacillus* species, which were incubated at 30°C (Ben Akacha, Michalak, Generalić Mekinić, et al. 2024; Ben Akacha et al. 2023).

#### 2.4.2 Determination of Inhibition Zones (IZ)

The agar‐well diffusion method was applied according to the method of Ellouze et al. (2024), with minor modifications. In this procedure, wells were made in Mueller‐Hinton agar plates (Biokar Diagnostics, Beauvais, France) using a sterile Pasteur pipette with a diameter of 6 mm. A fresh bacterial suspension, adjusted to 0.5 McFarland, was used to inoculate the plates. The plates were stored at 4°C for 2 h before incubating at 37°C for 18–24 h. The diameters of the inhibition zones were measured in mm, and all experiments were performed in triplicate (Ellouze et al. 2024).

#### 2.4.3 Determination of Minimum Inhibitory Concentration (MIC) and Minimum Bactericidal Concentration (MBC)

The MIC of EAPO was evaluated using the microdilution method, with slight modifications from the protocol outlined by Klubthawee et al. (2023). The EAPO was serially diluted in Mueller–Hinton broth containing bacteriological agar and dispensed into microplates. A standardised bacterial suspension was introduced into each well and incubated at 37°C for 18 h. After this period, tetrazolium salt was added, and the plates were further incubated for 2 h. The MIC was determined to be the lowest extract concentration, inhibiting bacterial growth and preventing the development of purple formazan. To determine the MBC, an aliquot (10 µL) from wells showing no visible bacterial growth was spread onto Mueller–Hinton agar and incubated at 37°C for 24 h. The MBC corresponded to the lowest concentration, which eliminated bacterial growth. All experiments were conducted in triplicate to ensure reliability.

### 2.5 Application of the Ethyl Acetate Extract of *P. odoratissimum* as Bio‐Preservatives

**FIGURE 1:**
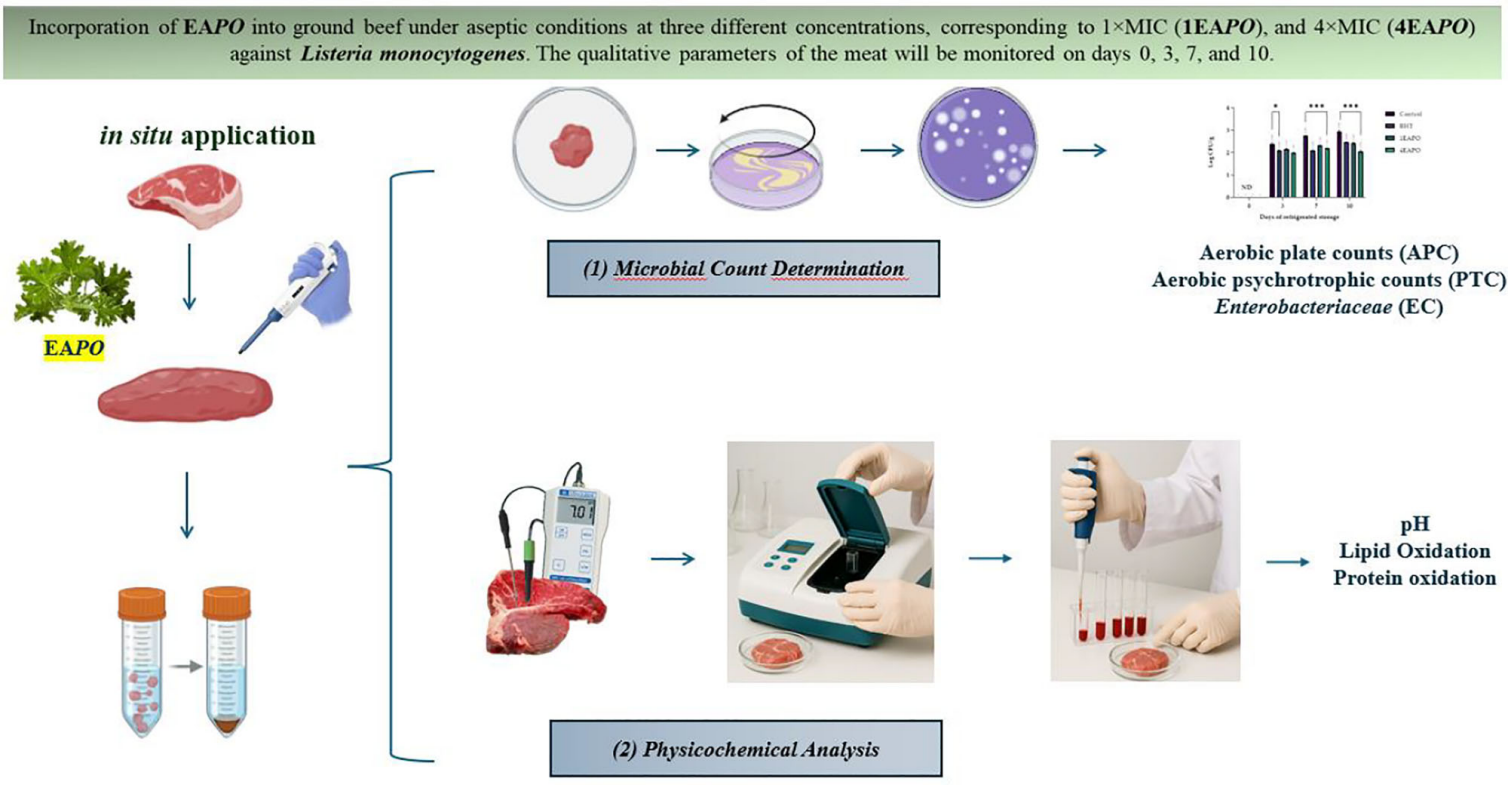
*In s* *itu* application of EAPO for ground beef preservation.

After determining the MIC and MBC of the EAPO, their potential as natural bio‐preservatives was assessed. The selected EAPOs were incorporated into the food matrix to evaluate their effectiveness in inhibiting bacterial growth and extending shelf life. The antimicrobial efficacy was monitored under controlled storage conditions, considering key microbiological and physicochemical parameters. This approach aimed to validate the practical application of essential oils as alternatives to synthetic preservatives in food preservation. To minimise potential confounding variables, all meat samples were sourced the same batch and supplier to reduce variability in pH, fat content, and initial microbial load. Samples were packed in high‐barrier polythene bags and stored at 4°C in dark conditions to avoid differences in light exposure. Storage took place in a controlled cold chamber with stable humidity and temperature. These measures ensured that the observed microbial and physicochemical changes were primarily due to the treatments applied and not to external factors (Figure 1.)

#### 2.5.1 Microbial Count Determinations

The microbiological analysis was conducted following the ISO 7218 standard after 14 days of storage at 4°C. Briefly, 25 g of the sample was homogenised in 225 mL of sterile 0.85% NaCl solution for 10 min. Serial decimal dilutions were then prepared for microbial enumeration. The microbiological analysis involved the enumeration of different bacterial groups to assess the microbial quality of the samples. Specific culture media and incubation conditions were used to ensure accurate quantification (Ben Akacha, Garzoli, et al. 2023; Taieb Bouteraa et al. 2024). The details of the methods applied are summarised in Table 1 below.

**TABLE 1:** Microbiological enumeration conditions.

Microbial group	Culture medium	Incubation conditions
Aerobic plate counts (APC)	Plate count agar	30°C for 48 h
Aerobic psychrotrophic counts (PTC)	Plate count agar	7°C for 10 days
*Enterobacteriaceae*	Violet red bile lactose agar	37°C for 24 h

#### 2.5.2 Physicochemical Analysis

Thiobarbituric acid reactive substances (TBARS) were measured as indicators of lipid oxidation. TBARS values were assessed based on the procedure described by Papastergiadis et al. (2012) where the absorbance of the meat samples was measured spectrophotometrically. The findings were expressed in milligrams of malondialdehyde (MDA) per kilogram of meat.

Metmyoglobin (MetMb) was quantified as described by Tang et al. (2004). In brief, 10 g of each meat sample was blended with 50 mL of phosphate buffer K_3_PO_4_ (pH 6.8, 0.04 M) followed by centrifugation (3000 × *g*, 30 min). After filtration, the absorbance of the filtrates was measured at 582, 525, 557, and 503 nm. The % of MetMb was quantified using the formula provided by Tang et al. (2004).

### 2.6 Statistical Analysis

All experiments were carried out in triplicate (*n* = 3) and values are reported as mean ± standard deviation (SD). Microbial counts were transformed to log_10_ CFU/g before analysis. Data were first checked for normality (Shapiro–Wilk test) and homogeneity of variance (Levene's test). For datasets meeting parametric assumptions, one‐way analysis of variance (ANOVA) was used to compare treatments at each storage time, followed by Tukey's HSD for pairwise comparisons. Significant differences between sample means at a 95% confidence level (*p < 0.05*).

For multivariate analysis, principal component analysis (PCA) and hierarchical cluster analysis (HCA) were conducted to evaluate the relationships between physicochemical and microbial parameters across different treatments and storage times. Ward's method and a Euclidean distance matrix were used for clustering to identify sample groupings based on their similarity in quality attributes.

All data were analyzed according to a completely randomized factorial design with two fixed factors: Treatment (four levels: Control, 0.01% BHT, 1% EAPO, 4% EAPO) and Storage Time (four levels: 0, 3, 7, and 10 days). Each parameter (microbial counts, pH, TBARS, MetMb, instrumental color, and sensory scores) was measured in three independent replicates per Treatment × Time combination.

$$\begin{array}{ccc} \mathrm{The}\;\mathrm{general}\;\mathrm{model}\;\mathrm{fitted}\;\mathrm{was}:\;\mathrm{Yijk} & = & \mu +\alpha i+\beta j+\left(\alpha \beta \right)ij\\ & & +\;\varepsilon ijk, \end{array}$$

where 𝜇 is the overall mean, 𝛼𝑖 is the effect of Treatment, 𝛽𝑗 is the effect of Storage Time, (𝛼𝛽)𝑖𝑗 is the interaction, and 𝜀𝑖𝑗𝑘 is the random error, assumed to be normally distributed with mean zero and constant variance.

Microbial counts were log_10_ transformed prior to analysis. Normality of residuals was assessed using the Shapiro–Wilk test and homogeneity of variances with Levene's test. When main effects or interactions were significant (*p < 0.05*), means were compared using Tukey's HSD post hoc test. Effect sizes (partial η^2^) were calculated to estimate the contribution of each factor.

### 2.7 Kinetic Analysis

To describe the time‐dependent deterioration of meat quality parameters (TBARS, MetMb, pH, color and sensory scores, APC/PTC/EC counts) we tested both zero‐order and first‐order kinetic models and selected the best model because of the coefficient of determination (*R* ^2^). For the first‐order model we assumed:

$$C_{t}=C_{0}e^{-kt}$$

which linearises to

$$\ln\;\left(C_{t}\right)=\ln\left(C_{0}\right)-kt.$$

The rate constant k (day^−1^) was obtained as the slope of the linear regression of ln(Ct) versus time (*t*). The half‐life (time to 50% change) was computed as:

$$t_{1}{/}_{2}=\ln2/k$$

All statistical analyses were performed using SPSS software (Version 26, IBM, USA) for ANOVA and Tukey's test, while XLSTAT (v.2014, Addinsoft, USA) was used for PCA and cluster analysis.

## 3 Results and Discussion

### 3.1 Chemical Analysis of *P. odoratissimum* Extracts

GC‐MS analysis identified 25 components differently distributed among the three extracts (Table 2). The compounds characterising the chemical profiles of the three extracts belonged to different chemical classes, such as terpenoids, steroid derivatives, and carboxylic acids and only four components, such as β‐citronellol, citronellyl isobutyrate, furopelargone A and 2‐phenylethyl tiglate, were present ubiquitously.

**TABLE 2:** Chemical volatile composition (percentages mean values ± standard deviation) of *P. odoratissimum* extracts.

N°	Component^1^	LRI^2^	LRI^3^	HEPO	DEPO	EAPO
1	trans‐rose oxide	1110	1115	1.2 ± 0.05	0.5 ± 0.03	—
2	cis‐rose oxide	1121	1128	0.8 ± 0.03	0.4 ± 0.02	—
3	isomenthone	1148	1152	1.2 ± 0.04	0.9 ± 0.04	—
4	β‐citronellol	1210	1212	31.9 ± 0.12	18.4 ± 0.12	55.8 ± 2.20
5	cis‐geraniol	1221	1227	2.2 ± 0.04	—	—
6	geranic acid	1351	1355	2.0 ± 0.05	—	5.8 ± 0.05
7	nerol acetate	1360	1363	—	0.3 ± 0.02	—
8	α‐copaene	1385	1392	—	0.4 ± 0.02	—
9	citronellyl isobutyrate	1510	1506	3.1 ± 0.04	1.0 ± 0.05	4.7 ± 0.04
10	geranyl isobutyrate	1512	1514	—	1.3 ± 0.05	6.5 ± 0.07
11	furopelargone A	1534	1540	1.4 ± 0.05	0.8 ± 0.02	2.9 ± 0.03
12	caryophyllene oxide	1571	1575	9.4 ± 0.06	—	—
13	2‐phenylethyl tiglate	1581	1587	5.6 ± 0.04	1.5 ± 0.04	5.5 ± 0.04
14	epicubenol	1610	1615	2.0 ± 0.03	—	—
15	δ‐cadinol	1628	1627	19.7 ± 0.11	—	—
16	geranyl angelate	1641	1648	13.4 ± 0.12	3.0 ± 0.05	—
17	nerolidol	1658	1656	3.1 ± 0.03	—	—
18	geranyl tiglate	1696	1695	3.0 ± 0.05	—	18.8 ± 0.21
19	santal camphor	1772	1774	—	1.6 ± 0.03	—
20	isopropyl myristate	1828	1831	—	2.2 ± 0.03	—
21	stigmastan‐6,22‐dien, 3,5‐dedihydro‐	2431	2437	—	3.8 ± 0.04	—
22	cholesta‐6,22,24‐triene, 4,4‐dimethyl‐	2570	2572	—	8.2 ± 0.07	—
23	stigmasta‐3,5‐diene	2712	2718	—	5.8 ± 0.04	—
24	spirosta‐5,7‐dien‐3‐ol	2861	2854	—	13.7 ± 0.21	—
25	β‐sitosterol acetate	3351	3357	—	36.2 ± 1.12	—
	**Sum**			100.0	100.0	100.0

^1^ The components are reported according to their elution order on a polar column.

^2^Linear retention indices measured on a polar column.

^3^Linear retention indices from literature.

“‐”—not detected; HEPO—hexane extract from *P. odoratissimum*; DEPO*—*dichloromethane extract from *P. odoratissimum*; EAPO*—*ethyl acetate extract from *P. odoratissimum*.

The values are presented as mean ± SD of three replicates.

EAPO was the extract with fewer compounds, where beta‐citronellol (55.8%) was the most abundant, followed by geranyl tiglate (18.8%). DEPO was distinguished from the other extracts as it was the only one in which a series of steroid derivatives had been detected and β‐sitosterol acetate (36.2%), β‐citronellol (18.4%), and spirosta‐5,7‐dien‐3‐ol (13.7%) reached the highest relative concentrations. On the other side, HEPO showed β‐citronellol (31.9%), δ‐cadinol (19.7%), and geranyl angelate (13.4%) as the main components.

The chemical profile of the EAPO extract shows a characteristic composition dominated by oxygenated monoterpenes and their ester derivatives, with β‐citronellol being the major constituent at 55.8%. This high proportion of β‐citronellol is consistent with previous reports highlighting its significant presence in plant extracts known for their aromatic and bioactive properties. β‐Citronellol is well documented for its antimicrobial, anti‐inflammatory, and antioxidant activities, suggesting that the EAPO extract may have similar pharmacological potential (Iqbal et al. 2024).

In addition to β‐citronellol, the extract contains significant amounts of geranyl tiglate (18.8%), geranyl isobutyrate (6.5%), geranic acid (5.8%), 2‐phenylethyl tiglate (5.5%), and citronellyl isobutyrate (4.7%). These compounds are mainly oxygenated monoterpenes and esters that contribute not only to the aromatic profile but also to the biological activity of the extract (Akçura et al. 2023).

The presence of geranic acid and various geranyl esters supports the potential antioxidant and anti‐inflammatory properties, as these compounds have previously been associated with such activities in related plant extracts (Moriki et al. 2025).

Compared to the other extracts analyzed (HEPO and DEPO), EAPO has a significantly higher concentration of these oxygenated monoterpenes, while significant amounts of sesquiterpenes and sterol derivatives are absent. This difference in composition may be due to different extraction methods affecting the solubility and yield of certain phytochemicals. The predominance of lighter, more volatile compounds in EAPO suggests that it is suitable for applications where aroma and bioactivity are paramount, such as in natural therapeutics or flavorings.

Overall, the chemical profile of the EAPO extract highlights its potential as a rich source of bioactive monoterpenoids and esters. Future studies should investigate the biological activities of this extract in vitro and In vivo to validate its therapeutic applications and explore its efficacy as a natural bioactive agent.

To determine the polyphenol content of the extracts, the Folin‐Ciocalteu reagent method was used, and the results are presented in Table 3 to estimate their richness in phenolic compounds.

**TABLE 3:** Phenolic compound content in different extracts of *P. odoratissimum*.

Extract	HEPO	DEPO	EAPO
**(µg EqGA/g)**	1.08 ± 1.26^b^	1.62 ± 1.16^b^	11.84 ± 5.76^a^

Polyphenol content expressed in µg gallic acid equivalent (EqGA) per gram of extract.

HEPO—hexane extract from *P. odoratissimum*; DEPO*—*dichloromethane extract from *P. odoratissimum*; EAPO*—*ethyl acetate extract from *P. odoratissimum*.

The values are expressed as mean ± SD (*n* = 3); samples with different letters (a, b) indicate significant differences (*p <* 0.05).

The total flavonoid content was quantified using the AlCl_3_ reagent, which forms acid‐resistant complexes with hydroxyl and neighboring ketone groups, as well as acid‐labile complexes with o‐dihydroxyl groups. The results of flavonoid quantification are presented in Table 4.

**TABLE 4:** Flavonoid content in different extracts of *P. odoratissimum*.

Extract	HEPO	DEPO	EAPO
**(µg EqQE/g)**	0.43 ± 0.02^b^	0.85 ± 0.14^b^	1.08 ± 0.01^a^

Flavonoid content expressed in mg quercetin equivalent (QE) per gram of extract.

HEPO—hexane extract from *P. odoratissimum*; DEPO*—*dichloromethane extract from *P. odoratissimum*; EAPO*—*ethyl acetate extract from *P. odoratissimum*.

The values are expressed as mean ± SD (*n* = 3); samples with different letters (a, b) indicate significant differences (*p* < 0.05).

Medicinal plants are rich sources of phenolic compounds, which are known for their significant contribution to antioxidant activity. These bioactive molecules, including flavonoids, phenolic acids, tannins, and anthocyanins, play crucial roles in protecting cells against oxidative stress and microbial attacks (Ben Hsouna et al. 2023; Ben Akacha et al. 2024). In this study, the total phenolic content of the ethyl acetate extract of *P. odoratissimum* was 11.84 µg GAE/g, while the total flavonoid content was 1.08 µg QE/g, indicating that polyphenols constitute the dominant group of antioxidant compounds in this plant. The lower number of flavonoids found compared to total polyphenols is in line with the fact that polyphenols include more than just flavonoids. They also contain tannins and stilbenes. The results align with previous research showing that medicinal plants contain diverse phenolic compounds, with more than 33 different phenolic molecules identified in various herbs.

### 3.2 Antioxidant Activity of *P. odoratissimum* Extracts

Given that phenolic content and flavonoids exhibit strong radical scavenging abilities, their presence in *P. odoratissimum* may explain its high antioxidant potential, as confirmed by the DPPH assay. The ethyl acetate extract of *P. odoratissimum* demonstrated the ability to scavenge free radicals, with an IC_50_ value of 10.65 ± 1.02 µg/mL. The herein investigated extract showed superior antioxidant activity compared with previous studies on *Pelargonium* sp. For example, reported IC_50_ values for DPPH scavenging in *Pelargonium* sp. ranged from 0.19 mg/mL in stems to 0.39 mg/mL in leaves, which are significantly higher than the value obtained in our study, indicating a greater radical scavenging capacity of the tested extracts. Similarly, in the ABTS test, the lowest IC_50_ values reported were 27.15 µg/mL and 28.11 µg/mL, both higher than the IC_50_ of our ethyl acetate extract, confirming its efficacy (Alonso et al. 2022). In addition, compared with aqueous extracts, which were characterised by the IC_50_ of 0.461 mg/mL, he herein tested ethyl acetate extract demonstrated significantly higher antioxidant capacity (Celi et al. 2024). Overall, the high phenolic content and antioxidant activity of *P. odoratissimum* suggest that its extracts could serve as valuable natural alternatives to synthetic antioxidants in food preservation and pharmaceutical applications. Further research should focus on isolating and characterising specific phenolic compounds better to understand their contributions to the observed bioactivity. Flavonoids remain essential due to their significant antimicrobial, anti‐inflammatory and antioxidant activity, which contributes to food preservation and safety (Pandey et Rizvi 2009). Similarly, phenolic compounds have attracted attention in the food industry because of their ability to inhibit microbial growth, delay lipid oxidation and extend shelf life. These properties are particularly important in meat preservation, where oxidative stability and microbial safety are critical. The strong antioxidant capacity of *P. odoratissimum* extracts therefore underlines their potential as natural additives in meat and other perishable foods (Rahman et al. 2021).

The antioxidant effect of EAPO is probably due to its high content of β‐citronellol, which is supported by geranic acid and geranyl/citronellyl esters (Iqbal et al. 2024). Monoterpenoid alcohols such as citronellol and the related compound geraniol have been reported to be consistently able to scavenge free radicals and attenuate lipid peroxidation in chemical and dietary models. Across multiple screening systems citronellol/geraniol are among the most active monoterpenes in DPPH/ABTS assays and in lipid systems (e.g., TBARS/β‐carotene bleaching), although absolute ranking may vary depending on the test matrix (Singh et al. 2012).

In vivo and cellular studies on the geraniol/citronellol family also show a strengthening of endogenous antioxidant defences (e.g., restoration of glutathione and antioxidant enzymes), confirming their contribution beyond direct free radical scavenging (Ben Akacha et al. 2024; Ben Hsouna et al. 2022).

Although specific data on geranyl/citronellol esters are more limited than on their parent alcohols, these lipophilic esters may act as reservoirs that effectively partition into lipid phases, where they can indirectly protect against oxidation or hydrolyse to active alcohols under certain conditions.

### 3.3 Antimicrobial Activity of *P. odoratissimum* Extracts

In recent years, plant‐derived bioactive compounds have garnered significant attention for their promising applications in human health, particularly as natural therapeutic agents. Driven by the need for safer and more sustainable alternatives, research efforts have intensified to uncover novel antimicrobial and antioxidant compounds that can replace synthetic counterparts. Among these natural candidates, medicinal plant extracts and their bioactive constituents have exhibited various potent biological activities, making them valuable prospects for pharmaceutical and food preservation applications (Vaou et al. 2021; S. Li et al. 2024). This study evaluates the antimicrobial potential of different *P. odoratissimum* extracts against human pathogenic strains. Antibacterial activity was assessed by measuring the inhibition zone diameter and observing the presence of a clear halo around the well, indicative of microbial growth inhibition. Furthermore, the MIC and the MBC were determined to quantify the antimicrobial efficacy of the extracts.

The antibacterial activity of *P. odoratissimum* extracts (hexane, dichloromethane, and ethyl acetate) was evaluated against Gram‐positive and Gram‐negative bacterial strains, using gentamicin as the reference antibiotic (Table 5). HEPO showed no inhibition against any of the strains tested, suggesting that its non‐polar constituents either lack antibacterial properties or are present in insufficient concentrations. In contrast, DEPO demonstrated moderate activity against Gram‐positive bacteria, with inhibition zones of 10 mm for *Staphylococcus aureus* and 8 mm for *Enterococcus faecalis*, but proved ineffective against Gram‐negative bacteria, probably due to the presence of an outer membrane limiting access to bioactive compounds. Among the extracts tested, EAPO showed the most potent antibacterial activity, particularly against Gram‐positive bacteria, with a zone of inhibition of 12 mm against *Listeria monocytogenes*. These results suggest that EAPO contains semi‐polar bioactive compounds with significant antimicrobial potential.

**TABLE 5:** Antibacterial activity of *P. odoratissimum* extracts against selected bacterial strains.

Bacterial strain	Inhibition zone diameter (mm)			
	HEPO	DEPO	EAPO	Gentamycin
**Gram‐positive**				
*Bacillus cereus* ATCC 14579	—	—	10 ± 0.02	10± 0.5
*Staphylococcus aureus* ATCC 25923	—	10 ± 0.01	11 ± 0.3	9.5 ± 0.1
*Enterococcus faecalis* ATCC 29212	—	8 ± 0.21	11.2 ± 0.25	8.7 ± 0.5
*Micrococcus luteus* ATCC 1880	—	—	8 ± 0.11	9.5 ± 0.5
*Listeria monocytogenes* ATCC 1911	—	—	12 ± 0.02	10 ± 0.4
**Gram‐negative**				
*Pseudomonas aeruginosa* ATCC 9027	—	—	9 ± 0.01	11 ± 0.5
*Escherichia coli* ATCC 25922	—	—	9 ± 0.3	12.5 ± 0.01
*Salmonella enterica* ATCC 43972	—	—	10 ± 0.21	10 ± 0.5

“‐”—no inhibition zone; gentamycin—reference antibiotic; HEPO—hexane extract of *P. odoratissimum*; DE—dichloromethane extract of *P. odoratissimum*; EA—ethyl acetate extract of *P. odoratissimum*.

The values are expressed as mean ± SD (*n* = 3).

Compared with extracts, EAPO's higher activity against Gram‐positive bacteria could be attributed to structural differences in bacterial walls, as Gram‐negative bacteria have an outer membrane that limits the penetration of antimicrobial agents (Akacha et al. 2022).

The antibacterial activity observed in EAPO is probably due to bioactive phenolic and flavonoid compounds, which are widely recognised for their antimicrobial properties. Previous studies have shown that ethyl acetate extracts from medicinal plants are often rich in polyphenols, flavonoids, and tannins, which exert their antibacterial effects by disrupting bacterial walls, inhibiting key enzymes and interfering with microbial metabolism (Bittner Fialová et al. 2021). The significant inhibition observed against *Listeria monocytogenes* and *Staphylococcus aureus* suggests the presence of flavonoids such as quercetin and kaempferol derivatives, known for their potent antibacterial activity against Gram‐positive bacteria. In addition, phenolic acids, notably gallic acid and caffeic acid, frequently found in plant extracts, could contribute to the antibacterial activity observed by inducing oxidative stress in bacterial cells, leading to membrane damage and metabolic disruption (Todorov et al. 2025).

As well, the antibacterial activity of EAPO is mechanistically consistent with the literature on monoterpenes, disrupting bacterial membranes, reducing ion gradients, and altering biofilms. Among the components of EAPO, β‐citronellol is a well‐documented antibacterial monoterpenoid alcohol comparative studies identify citronellol as one of the most effective components against Gram‐positive and Gram‐negative species through membrane damage and K⁺ leakage (Y. Li et al. 2024).

Geranic acid (the trans isomer of 3,7‐dimethyl‐2,6‐octadienoic acid) also shows antibacterial effects; in choline‐geranic acid systems, the destruction efficiency correlates with the geranic acid content and is attributed to cell envelope disruption (Laird et al. 2023).

The geranyl esters present in EAPO (geranyl tiglate, geranyl isobutyrate) are less well studied than the alcohol, but available reports indicate measurable activity against *Escherichia coli* and other bacteria, consistent with the more general observation that geraniol‐derived esters retain membrane active properties; nevertheless, more targeted MIC work is still required for these specific esters (Chacón et al. 2019). Finally, the genus *Pelargonium* itself is rich in antibacterial monoterpenes (citronellol‐ and geraniol‐dominant chemotypes), and numerous studies show robust activity of *Pelargonium* oils against *Staphylococcus aureus* (including MRSA), supporting the plausibility that EAPO's major monoterpenoids are primary drivers of its antimicrobial effect in meat (Bigos et al. 2012).

These results highlight the potential of *P. odoratissimum* as a source of antibacterial compounds, particularly in its ethyl acetate fraction, warranting further studies to isolate and characterise the active molecules, determine their mechanism of action, and explore their potential applications in food preservation and pharmaceutical formulations.

Inhibition zone results revealed that EAPO had the strongest activity, suggesting that the active compounds responsible for microbial inhibition are probably more soluble in ethyl acetate. MIC and MBC data confirmed these results (Table 6). The MBC/MIC ratio was ≤2 for all strains tested, indicating that EAPO has a bactericidal rather than bacteriostatic effect.

**TABLE 6:** MIC and MBC of EAPO extract against selected bacterial strains.

Bacterial strain	MIC (mg/mL)	MBC (mg/mL)	MBC/MIC	Interpretation
**Gram‐positive**				
*Bacillus cereus* ATCC 14579	3.75 ± 0.76	3.75 ± 0.25	1	Bactericidal
*Staphylococcus aureus* ATCC 25923	5.01 ± 0.04	6.25 ± 0.04	1.24	Bactericidal
*Enterococcus faecalis* ATCC 29212	2.5 ± 0.00	3.75 ± 0.01	1.5	Bactericidal
*Micrococcus luteus* ATCC 1880	7.5 ± 0.53	7.5 ± 0.21	1	Bactericidal
*Listeria monocytogenes* ATCC 1911	6.25 ± 0.03	6.25 ± 0.21	1	Bactericidal
**Gram‐negative**				
*Pseudomonas aeruginosa*c ATCC 9027	3.12 ± 0.65	3.12 ± 0.05	1	Bactericidal
*Escherichia coli* ATCC 25922	2.5 ± 0.01	3.75 ± 0.21	1.5	Bactericidal
*Salmonella enterica* ATCC 43972	3.75 ± 0.76	3.75 ± 0.21	1	Bactericidal

The values are expressed as mean ± SD (*n* = 3).

The bactericidal effect of *P. odoratissimum* extract can be attributed to its rich composition of secondary metabolites, in particular monoterpenes (e.g., β‐citronellol, geranic acid, and geranyl esters) and polyphenolic compounds. Monoterpenes are known to integrate into bacterial lipid bilayers, leading to loss of membrane integrity, leakage of intracellular contents, and breakdown of the proton motive force, while polyphenols exert complementary effects through enzyme inhibition, metal ion chelation, and modulation of oxidative stress. Such multimodal mechanisms are favourable as they reduce the likelihood of bacterial resistance development. The MIC values obtained in our study are consistent with previous reports documenting inhibition levels of 500–1000 µg/mL against *Staphylococcus aureus* (including methicillin‐resistant strains) (Patel et al. 2009). These results emphasise the potential of *P. odoratissimum* as a natural strategy to combat antibiotic‐resistant pathogens, which is urgently needed in both clinical and food microbiology (Akacha et al. 2022).

Importantly, bactericidal compounds differ from bacteriostatic agents in that they destroy bacterial populations rather than merely suppressing their growth, making them desirable for food preservation and therapeutic applications where complete elimination of microbes is required. Our data are consistent with the findings of Fernandez‐Soto et al. (2023) who reported that extracts of *Cinnamomum* spp. and *P. odoratissimum* exhibit bactericidal effects, as evidenced by a minimum bactericidal concentration (MBC) to MIC ratio below 4. This parameter is widely accepted as a characteristic for categorising agents as bactericidal, placing EAPO alongside traditional antibiotics in its ability to directly kill bacteria. The decision to focus on the ethyl acetate fraction (EAPO) was justified by its improved antimicrobial efficacy compared to other solvent fractions. Ethyl acetate preferentially extracts flavonoids, coumarins, and phenolic acids, which have been consistently associated with strong antibacterial activities. Furthermore, ethyl acetate is less toxic and more biocompatible than solvents such as methanol, chloroform, or hexane, enhancing its suitability for applications in food safety and pharmaceutical formulations. Apart from its safety advantages, the selectivity of ethyl acetate extraction also leads to a concentration of medium‐polarity compounds that act synergistically with monoterpenes and can enhance the overall bactericidal effect (Gopčević et al. 2022).

From an application perspective, EAPO is a promising natural preservative that could be integrated into active food packaging systems, edible coatings, or directly into meat and dairy products to suppress spoilage and pathogenic microorganisms. Such applications would meet current consumer demand for “clean‐labelled” products and regulatory pressure to reduce the use of synthetic preservatives. In clinical contexts, EAPO could also be explored as an adjuvant to conventional antibiotics, as plant extracts have been reported to often act synergistically with existing antimicrobials, reducing the doses required and potentially reversing resistance phenotypes.

Overall, the strong bactericidal potential of EAPO, combined with its relative safety and selective extraction profile, positions it as a compelling candidate for development as a natural antimicrobial alternative to synthetic preservatives and conventional antibiotics. Its integration into food systems and healthcare will require a multidisciplinary approach, combining chemistry, microbiology, toxicology, and formulation science to unlock its full potential.

### 3.4 Application of EAPO As a Ground Beef Meat Bio‐Preservative

#### 3.4.1 Microbiology Analysis

##### 3.4.1.1 Aerobic Plate Count (APC)

**FIGURE 2:**
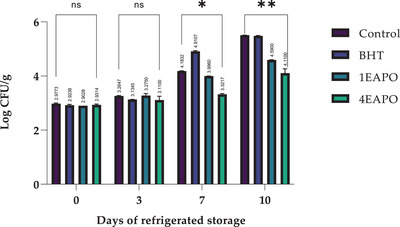
Evolution of APC during 10 days of refrigerated storage. Control—untreated sample; BHT—sample treated with 0.01% BHT; 1EAPO and 4EAPO—samples treated with EAPO at 1× and 4× the MIC against *Listeria monocytogenes*, respectively. The values are presented as mean ± standard deviation (SD) of three replicates. (ns) non‐significant, ***p ≤ 0.001, **p ≤ 0.01, *p ≤ 0.05 for the same concentration are significantly different.

The results of this study highlight the potential of EAPO as an effective bio‐preservative for ground beef due to its strong antibacterial and antioxidant properties. The observed reduction in APC, particularly at 4EAPO, underlines its ability to inhibit bacterial growth and extend meat shelf life (Figure 2).

Based on previous research, the antimicrobial properties of the extract of PO (EAPO) are due to methyl eugenol and citronellol. These bioactive compounds work by damaging the bacterial cell membrane, which in turn reduces their viability. Additionally, these compounds are potent antioxidants that can prevent lipid oxidation, a major cause of meat spoilage. For frozen ground meat, a proposed standard limit for APC is 5 × 10^5^ CFU/g. For fresh or frozen meats, a different standard sets a limit of 5 × 10^6^ CFU/g (Liu et al. 2023).

EOPO's dual action—preventing microbial contamination and oxidative degradation—makes it a promising alternative to synthetic preservatives, which can pose toxicity problems.

Additionally, other *Pelargonium* sp., including *Pelargonium graveolens*, have been shown to possess broad‐spectrum antimicrobial activity, with some studies reporting fungi toxic properties superior to synthetic fungicides (Celi et al. 2024). The rich composition of *Pelargonium* extracts, which includes monoterpenes, sesquiterpenes, tannins, and flavonoids, contributes to their potent bioactivity (Ben Hsouna, Ben Akacha, et al. 2024). These compounds have been widely recognised for their role in inhibiting foodborne pathogens, including *Listeria monocytogenes* and *Salmonella enterica*, which are major concerns in the meat industry. Furthermore, EAPO is a promising candidate for incorporation into food preservation strategies, ensuring safety and extended shelf life in meat products.

##### 3.4.1.2 Psychotropic Count (PTC)

Controlling PTC bacteria in meat is essential to guarantee food quality and safety. These bacteria, capable of growing at refrigeration temperatures, significantly cause spoilage in chilled and frozen meat products (Saucier 2016). Their metabolic activities lead to undesirable texture, odour, and flavour changes, considerably reducing shelf life (Ben Akacha et al. 2024). Although their activity is greatly reduced in frozen conditions, PTC bacteria can still be present. When the meat is thawed, these bacteria can resume growth and cause spoilage. Studies on frozen beef have shown PTC counts in the range of 1.5–3.71 log CFU/g (Zhu et al. 2022).

In addition, many PTC species produce extracellular enzymes, such as lipases and proteases, which accelerate meat spoilage by breaking down fats and proteins. In addition to spoilage problems, some PTC bacteria are food‐borne pathogens, posing a health risk to consumers (Aziz et Karboune 2018). Effective monitoring of these microorganisms helps to improve hygiene control in meat production, identify sources of contamination, and implement the necessary interventions. In addition, maintaining strict temperature control is essential to limit their growth and preserve meat quality. By systematically monitoring PTC bacteria, the meat industry can improve food safety, reduce waste, and ensure compliance with regulatory standards.

**FIGURE 3:**
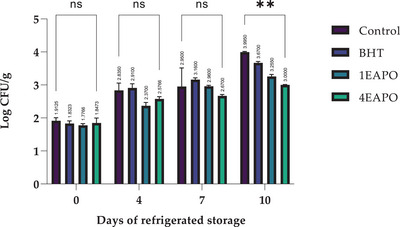
Evolution of PTC in refrigerated storage. Control—untreated sample; BHT—sample treated with 0.01%BHT; 1EAPO and 4EAPO—samples treated with EAPO at 1× and 4× the MIC against *Listeria monocytogenes*, respectively. The values are presented as mean ± SDs of three replicates. ns—non‐significant, ***p ≤ 0.001, **p ≤ 0.01, *p ≤ 0.05 for the same concentration are significantly different.

The results of this study highlight the potential of EAPO as a natural preservative for beef minced meat. The data revealed that the ethyl acetate extract effectively reduced the growth of psychotropic microorganisms in a dose‐dependent manner, with the highest concentrations showing the most significant antimicrobial effects. This suggests EAPO possesses strong preservative properties, making it a promising natural alternative to synthetic preservatives such as BHT. Using ethyl acetate as an extraction solvent further enhances these properties, effectively isolating the bioactive compounds responsible for antimicrobial activity (Figure 3).

The use of medicinal plants, such as *P. odoratissimum*, in food preservation is attracting increasing attention due to rising consumer demand for natural and clean‐label products. Plant‐derived alternatives are considered safer and more sustainable. Rich in bioactive compounds including phenolic acids, terpenoids, and flavonoids medicinal plants exhibit a wide range of antimicrobial, antioxidant, and anti‐inflammatory activities.

##### 3.4.1.3 Enterobacteriaceae Count (EC)

Controlling the quantity of *Enterobacteriaceae* is an essential element of food protection, as this bacterial circle of relatives includes spoilage organisms and ability food‐borne pathogens. Enterobacteria, consisting of *Escherichia coli*, *Salmonella*, *Klebsiella* and *Enterobacter*, are widely diagnosed as indicators of the hygiene, protection and microbial high quality of meal products (Mladenović et al. 2021). Their presence and increase in meals may signal contamination, insufficient processing or negative storage situations, raising the hazard of spoilage and meals‐borne illness. Studies on frozen beef meat showed *Enterobacteriacae* counts were in the range of 1.5–3.71 log CFU/g (Pérez Chabela et al. 1999).

Regarding meal upkeep, controlling EC is critical to guarantee product protection and amplify shelf life (Ben Akacha, Garzoli, et al. 2023). These bacteria can proliferate swiftly under favorable conditions, such as inadequate refrigeration or poor handling, and contribute to spoilage via enzymatic degradation of proteins and carbohydrates, resulting in unsightly odors, discolorations, and textural modifications. In addition, specific individuals of the EC, appreciably *Salmonella* and *Shigella*, are the foremost pathogens responsible for food‐borne epidemics, making their detection and quantification a critical issue of meals safety management (Bintsis 2017).

**FIGURE 4:**
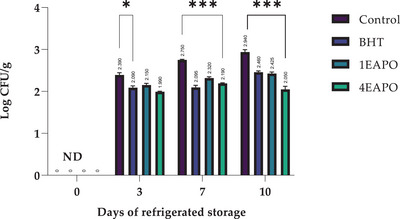
*Enterobacteriaceae* growth in refrigerated Storage. Control—untreated sample; BHT—sample treated with 0.01%BHT; 1EAPO and 4EAPO—samples treated with EAPO at 1× and 4× the MIC against *Listeria monocytogenes*, respectively. The values are presented as mean ± SD of three replicates. ns—non‐significant, ***p ≤ 0.001, **p ≤ 0.01, *p ≤ 0.05 for the same concentration are significantly different.

The data presented in Figure 4 reflects the development of EC in ground meat stored at 4°C for 14 days of storage. The control sample showed a steady increase in microbial loads from 1.98 ± 0.01 to 3.98 ± 0.08 in 10 days, indicating natural microbial spread without an antimicrobial agent.

Samples treated with EAPO extracts showed an essential preventive effect. In the lowest concentration (1EAPO), microbial growth was reduced, reaching 2.94 ± 0.06 today, 10, much lower than control and BHT‐treated samples. The highest concentration (4EAPO) showed the most potent antimicrobial activity, with the number of microbes falling from 1.89 ± 0.07 at only 2.74 ± 0.04 on day 0 to only 2.74 ± 0.04 in 10 days.

These results suggest that EAPO extracts have significant antimicrobial properties and effectively reduce microbial spread in a concentration‐dependent manner. The extracted items from BHT emphasise their ability as a natural alternative to synthetic antimicrobial agents. They were related to other studies using ethyl acetate extracts from medicinal plants. They attracted significant attention to food protection due to the rich structure of bioactive compounds with antimicrobial and antioxidant properties. For example, the extracts of ethyl acetate from the stems and leaves of *Astragalus*’s membrane have shown remarkable antibacterial activity against foodborne pathogens, which suggests their ability as a natural protector in food applications (Guo et al. 2022). Similarly, the ethyl acetate extracts from *Pongamia pinnata* blades showed more antibacterial effects than the extracts of chloroform and methanol, which highlight their impact in interrupting microbial growth (Nyalo et al. 2023).

These results suggest that EAPO possesses significant antimicrobial properties, effectively slowing microbial proliferation in a concentration‐dependent manner. Further research should focus on identifying the specific bioactive compounds responsible for this antimicrobial effect and evaluating their effectiveness in different food matrices.

#### 3.4.2 Biochemical Analysis

##### 3.4.2.1 pH

Monitoring the pH throughout meat storage is vital for assessing its quality, safety, and shelf life. pH is a key indicator of microbial activity and spoilage in meat. As the pH of meat increases due to microbial growth and enzymatic activity, it creates an environment more conducive to the proliferation of spoilage microorganisms and pathogens. Elevated pH levels can result in undesirable texture, color, and odor changes indicative of meat deterioration (Karanth et al. 2023). Regularly monitoring pH makes it possible to track the progression of spoilage and determine the effectiveness of preservation methods. Moreover, pH can also impact the product's overall sensory properties and consumer acceptability. Maintaining optimal pH levels helps ensure that meat remains safe, fresh, and of high quality throughout its storage and display period, ultimately contributing to prolonged shelf life and reducing waste (Ben Hsouna et al. 2022).

Table 7 shows the evolution of pH in ground beef stored at 4°C for 14 days for different treatment groups. In the control group, pH increased from 5.37 ± 0.02 on day 0 to 6.89 ± 0.24 on day 10. This steady increase in pH can be attributed to microbial activity and protein degradation, which often lead to increased pH in meat during storage. In the BHT‐treated sample, pH ranges from 5.27 ± 0.17 on day 0 to 6.42 ± 0.18 on day 10. Although BHT has some preservative properties, its effect on slowing pH changes was not as pronounced as expected, indicating that microbial and enzymatic activity still contributed to the pH increase.

**TABLE 7:** Effect of EAPO on pH values of raw minced meat beef during storage at 4°C.

Samples	Days of refrigerated storage			
	0	3	7	10
**Control**	5.37 ± 0.02^aA^	5.88 ± 0.02^bAB^	6.20 ± 0.06^bB^	6.89 ± 0.24^dBC^
**BHT**	5.27 ± 0.17^aA^	5.70 ± 0.06^bAB^	6.01 ± 0.01^abB^	6.42 ± 0.18^cBC^
**1EAPO**	5.23 ± 0.24^aA^	5.71 ± 1.021^aA^	5.98 ± 0.91^bAB^	6.12 ± 0.10^cB^
**4EAPO**	5.24 ± 0.01^aA^	5.28 ± 0.07^aAB^	5.70 ± 0.14^abAB^	5.88 ± 0.03^bBC^

Control—untreated sample; BHT—sample treated with 0.01% BHT; 1EAPO and 4EAPO—samples treated with EAPO at 1× and 4× the MIC against *Listeria monocytogenes*, respectively.

The values are presented as mean ± SD of three replicates; a–d: mean values with in all the samples not followed by a similar letter in the same column varied significantly (*p* < 0.05).

The pH values showed varied results for samples treated with different concentrations of EAPO extract. Sample 3 showed a similar trend to the control, with pH rising from 4.23 ± 0.24 to 6.12 ± 0.10. This indicates that the lower concentration of EAPO slightly slowed the increase in pH compared with the control.

Overall, the data suggest that treatment with the ethyl acetate extract of *P. odoratissimum* can slow the pH rise in ground beef, with higher concentrations being the most effective. This could indicate that EAPO has potential as a natural preservative in meat products, inhibiting microbial growth and retarding spoilage.

##### 3.4.2.2 Lipid Peroxidation

Monitoring TBARS is crucial in meat conservation as it is a reliable indicator of lipid oxidation, significantly impacting meat quality and shelf life (Papastergiadis et al. 2012). Elevated TBARS values are associated with increased oxidative degradation of lipids, leading to undesirable changes in flavor, color, and texture, thereby reducing consumer acceptance. By quantifying TBARS, producers can assess the effectiveness of preservation techniques, such as high‐pressure treatments, and implement strategies to mitigate oxidative rancidity, ensuring the delivery of high‐quality meat products to consumers (Ellouze et al. 2024).

Table 8 shows that the control group exhibited the highest TBARS values at the end of the storage period (1.30 ± 0.21 mg MDA‐eq/kg at day 10), confirming the absence of antioxidant protection. In contrast, samples treated with BHT, 1EAPO, and 4EAPO showed significantly lower TBARS values throughout storage, demonstrating their effectiveness in delaying lipid oxidation.

**TABLE 8:** Lipid oxidation (TBARS values) during refrigerated storage (4°C) under different treatments.

Samples	Days of storage at 4°C			
	0	3	7	10
Control	0.13 ± 0.89^bA^	0.53 ± 0.66^cB^	0.97 ± 0.17^bBC^	1.30 ± 0.21^cCD^
BHT	0.13 ± 0.52^aA^	0.40 ± 0.01^aA^	0.87 ± 0.16^bB^	1.08 ± 0.21^cC^
1EAPO	0.12 ± 0.42^aA^	0.38 ± 0.24^cB^	0.79 ± 0.15^abB^	0.90 ± 0.22^bcC^
4EAPO	0.15 ± 0.02^aA^	0.21 ± 0.01^cB^	0.61 ± 0.11^abB^	0.77 ± 0.92^bcC^

Control—untreated sample; BHT—sample treated with 0.01% BHT; 1EAPO and 4EAPO—samples treated with EAPO at 1× and 4× the MIC against *Listeria monocytogenes*, respectively.

The values are presented as mean ± SD of three replicates.

a–d: mean values with in all the samples not followed by a similar letter in the same column varied significantly (*p* < 0.05).

Among the natural preservatives, 2EAPO exhibited the most potent antioxidant activity, maintaining the lowest TBARS values, followed by 1EAPO and BHT. This suggests that 4EAPO has superior lipid oxidation inhibition, potentially due to a higher concentration of bioactive compounds. The results confirm that extract (EAPO) can effectively preserve lipid stability in meat, offering a promising natural alternative to synthetic antioxidants like BHT.

During storage, natural extracts can help keep TBARS levels in check. Studies have shown that adding natural extracts with antioxidant functions can decrease lipid oxidation, lowering TBARS values in raw and cooked meat products. Specifically, rosemary extract has been shown to reduce TBARS value in beef (Georgantelis et al. 2007). Natural extracts can improve antioxidant capacity and inhibit microbial growth, extending the shelf life and delaying the deterioration of sensory properties in meat. The effectiveness of phyto‐extracts in inhibiting lipid oxidation has also been demonstrated, with certain combinations leading to the lowest TBARS values in meat products. However, TBARS values tend to increase with storage time, although natural extracts can mitigate this increase. Predictive models are being developed to understand and compare the effects of different plant extracts on lipid oxidation in meat.

##### 3.4.2.3 Protein Oxidation

Determining MetMb oxidation during refrigerated storage is crucial for maintaining meat products' visual appeal and perceived freshness (Nethra et al. 2023). Metmyoglobin, a brown pigment formed when myoglobin oxidises, causes meat to appear discoloured, leading consumers to associate it with spoilage and reduced quality. Producers can assess the effectiveness of preservation methods and storage conditions by closely tracking metmyoglobin levels, ensuring optimal color retention and extending shelf life (Nethra et al. 2023). This practice helps maintain product quality and plays a significant role in influencing purchasing decisions, as consumers often rely on meat color as an indicator of freshness.

The data (Table 9) shows that EAPO treatments effectively slow deterioration, probably due to their potent antioxidant and antimicrobial properties. The control sample showed the fastest deterioration, underlining the need for conservation strategies. While BHT treatment provided some protection, it was less effective than EAPO treatments. Notably, 4EAPO showed the slowest degradation, with a final value of 12.67 ± 0.05%, compared with 31.32 ± 0.05% in the control, confirming a dose‐dependent protective effect.

**TABLE 9:** Metmyoglobin oxidation (%) in meat samples during refrigerated storage at 4°C.

Samples	Days of storage at 4°C			
	0	3	7	10
Control	5.42 ± 0.42^aA^	16.31 ± 0.41^dB^	20.67 ± 0.56^dC^	31.32 ± 0.05^cD^
BHT	6.88 ± 0.21^bA^	10.15 ± 0.41^cdB^	16.58 ± 0.56^cdC^	24.60 ± 0.05^bcD^
1EAPO	6.20 ± 0.21^bA^	11.65 ± 0.41^cdB^	13.42 ± 0.56^cdC^	17.84 ± 0.05^bcD^
4EAPO	6.47 ± 0.21^bA^	8.32 ± 0.41^cdB^	10.59 ± 0.56^cdC^	12.67 ± 0.05^bcD^

Control—untreated sample; BHT—sample treated with 0.01% BHT; 1EAPO and 4EAPO—samples treated with EAPO at 1× and 4× the MIC against *Listeria monocytogenes*, respectively.

The values are presented as mean ± SD of three replicates; a–d: mean values with in all the samples not followed by a similar letter in the same column varied significantly (*p* < 0.05).

While EAPO showed dose‐dependent antioxidants and antimicrobial effects in this study model system, its practical replacement of synthetic antioxidants (e.g., BHT) requires rigorous techno‐economic and safety justification (Fernandez‐Soto et al. 2023). Natural extracts are generally associated with higher raw material and processing costs and exhibit greater batch‐to‐batch variability. Therefore, pilot‐scale extraction yield, solvent recovery, and cost per active unit need to be quantified to assess cost efficiency. Process intensification (optimised solvent selection, concentration, and encapsulation technologies) can reduce effective consumption and sensory impact but increases formulation complexity and cost. For regulatory approval, a clear compositional specification, stability data, intended use levels and exposure estimates must be submitted to the competent authority. From a safety perspective, *Pelargonium* extracts require toxicological profiling and targeted screening for potentially genotoxic constituents (e.g., methyleugenol and related phenylpropanoids), followed by margin‐of‐exposure (MOE) or acceptable‐intake calculations at proposed use levels; if such compounds are present, compositional control or selective fractionation will be necessary. In short, EAPO is promising, but commercialization will depend on demonstrated manufacturing consistency, favorable unit economics at pilot scale, robust safety data, and formulation strategies that preserve efficacy while meeting regulatory and sensory constraints.

#### 3.4.3 Statistical Analysis

##### 3.4.3.1 Principal Component Analysis (PCA)

**FIGURE 5:**
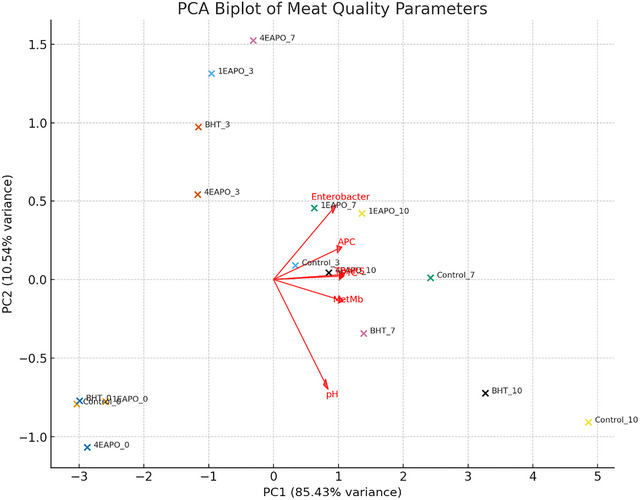
Principal component analysis of meat quality parameters under different antioxidant treatments during storage. Control‒untreated sample; BHT‒sample treated with 0.01% BHT; 1EAPO and 4EAPO‒samples treated with EAPO at 1× and 4× the MIC against *Listeria monocytogenes*, respectively, during 10 days of storage at 4°C.

**FIGURE 6:**
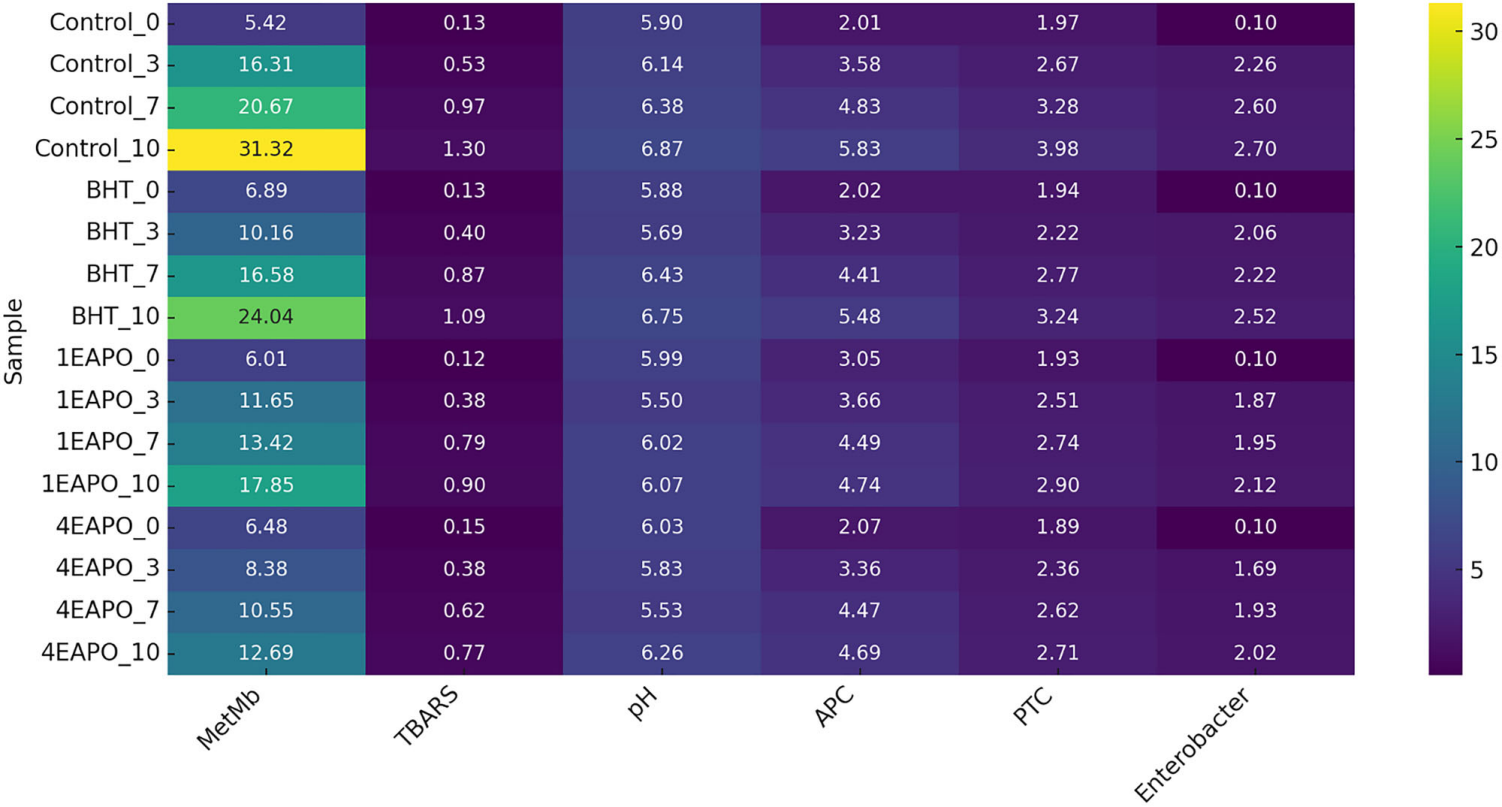
Heat map visualisation of oxidation and microbial growth in meat. The scale bar on the right side of the heatmap represents the relative magnitude of each measured parameter. Low values are shown in dark purple to blue shades, intermediate values appear in green to yellow tones, while the highest values are highlighted in bright yellow. As each parameter (MetMb, TBARS, pH, APC, PTC, and Enterobacteriaceae) has its own numerical range, the color intensity is scaled independently, ensuring that the gradient accurately reflects the variation within each parameter.

The principal component analysis (PCA) explained 95.11% of the total variance in meat quality parameters, with PC1 (87.64%) capturing the major differences related to storage progression and PC2 (7.47%) accounting for minor variations (Figure 5). Along PC1, a clear temporal trend was observed, where control samples shifted strongly from the left (day 0) to the right (day 10), indicating pronounced deterioration of quality attributes over time. In contrast, BHT‐ and *P. odoratissimum* (1EAPO and 4EAPO) exhibited a slower shift along PC1, reflecting their protective effect in maintaining meat quality during storage. Notably, samples treated with the higher *P. odoratissimum* concentration (4EAPO) clustered closer to the initial fresh state compared to controls, suggesting enhanced efficacy in delaying spoilage. PC2 contributed to separating treatments based on more subtle differences, likely related to the specific antioxidant action of BHT and essential oils. Overall, the PCA highlights that natural treatments, particularly at higher doses, preserved meat quality more effectively than the control, with an effect comparable to the synthetic antioxidant BHT (Figure 6)

Several studies have used PCA to assess the impact of natural antioxidants on meat quality, focusing on parameters such as lipid oxidation. Chemometric modeling, including PCA, linked antioxidants, neutral lipid fatty acids, and flavor components in chicken breasts (Ben Akacha et al. 2023; Ellouze et al. 2024). Conversely, the PCA results provided insight into the relationships between antioxidant treatments and the meat quality, helping to understand how natural antioxidants influence the sensory attributes of meat products (Zhang et al. 2022). These studies demonstrate that PCA is a valuable tool for elucidating the effects of natural antioxidants on various meat quality parameters, including lipid oxidation, stability profiles. The application of PCA facilitates the identification of patterns and correlations in complex data sets, allowing us to better understand how natural antioxidants can improve meat quality and extend shelf life.

##### 3.4.3.2 Heat Maps

The heatmap provides an integrative overview of the evolution of physicochemical and microbiological parameters during refrigerated storage of meat, highlighting the differences between untreated control samples and those treated with BHT or *P. odoratissimum* (EAPO). In the control group, a progressive deterioration was observed, as reflected by sharp increases in MetMb and TBARS values, indicative of protein and lipid oxidation, respectively. These changes are consistent with the expected oxidative instability of meat during storage and are in line with earlier reports showing that oxidation processes accelerate discoloration and rancidity development (Nabizadeh et al. 2024).

Treatment with antioxidants, both synthetic (BHT) and natural (EAPO), attenuated these changes as shown by the lower intensity of the heat map values compared to the control. In particular, the 4% EAPO treatment showed the most pronounced protective effect by limiting the accumulation of metmyoglobin and secondary lipid oxidation products. This effect is probably due to the high phenolic content of the oil, which can donate hydrogen atoms to neutralise free radicals and interrupt oxidative chain reactions. Interestingly, noticeable antioxidant activity was also obtained with 1 EAPO, although the effect was concentration‐dependent, with higher efficacy observed at 4%.

The microbiological indicators (APC, PTC and Enterobacteriaceae) also showed different patterns in the heat map. The control samples showed rapid bacterial proliferation consistent with the increase in pH values, typically reflecting microbial metabolism and protein degradation (Yang et al. 2025). Both BHT and EAPO suppressed microbial growth; however, 4% EAPO achieved the strongest inhibition and kept microbial counts at a lower level throughout storage. This antimicrobial activity may be related to the disruption of bacterial cell membranes and enzyme systems by plant extract constituents such as terpenes and phenolic compounds, a mechanism that has been widely reported in the literature (Angane et al. 2022)

The heatmap visualization thus proves to be a powerful tool for simultaneously assessing multiple quality indicators and detecting treatment‐related trends. It highlights the multidimensional benefits of EAPO in extending meat shelf life by mitigating both oxidative and microbial spoilage pathways. From a broader perspective, the results strengthen the case for the application of essential oils as natural alternatives to synthetic preservatives in meat products, aligning with consumer demand for clean‐label foods.

##### 3.4.3.3 Kinetic Model Selection

Rationale for first‐order selection and interpretation. First‐order kinetics were chosen because the time courses of several quality indices (TBARS, MetMb, and microbial counts) exhibited approximately exponential behaviors (linear ln‐plots and higher *R* ^2^ for the first‐order fit compared with zero‐order), indicating that the instantaneous deterioration rate was approximately proportional to the remaining “goodness” of the parameter. The first‐order model therefore provides a simple, commonly used empirical description of oxidative and spoilage processes in complex foods and allows direct comparison between treatments via the rate constant *k* and half‐life t_1/2_. This selection and the resulting *k*/t_1/2_ values are reported in Table 10.

**TABLE 10:** Comparison of rate constants, half‐life, and *R* ^2^ values for various treatments on meat quality parameters.

Parameter	Treatment	Rate constant (k) (day^−1^)	*R* ^2^	Half‐life (t_1/2_) (days)
TBARS (MDA‐eq/kg)	Control	0.22	0.98	3.15
	BHT	0.15	0.96	4.62
	1EAPO	0.13	0.97	5.33
	4EAPO	0.11	0.99	6.30
MetMb (%)	Control	0.25	0.97	2.77
	BHT	0.18	0.95	3.85
	1EAPO	0.14	0.96	4.95
	4EAPO	0.12	0.98	5.78
pH	Control	0.08	0.92	8.66
	BHT	0.06	0.93	11.55
	1EAPO	0.05	0.94	13.86
	4EAPO	0.04	0.96	17.33
EC counts (log CFU/g)	Control	0.28	0.98	2.47
	BHT	0.22	0.96	3.15
	1EAPO	0.19	0.97	3.65
	4EAPO	0.16	0.98	4.33

The values are presented as mean ±SD of three replicates.

a–d: mean values with in all the samples not followed by a similar letter in the same column varied significantly (*p* < 0.05).

Control—untreated sample; BHT—sample treated with 0.01% BHT; 1EAPO and 4EAPO—samples treated with EAPO at 1× and 4× the MIC against *Listeria monocytogenes*, respectively.

For each parameter the measured values at the sampling times (0, 3, 7, 10 days) were log‐transformed and fitted by linear regression against time. The negative of the regression slope was taken as *k* (day^−1^) and t_1/2_ was calculated as ln (2)/*k*. The goodness of fit (*R* ^2^) was inspected and used to choose between zero and first‐order fits.

###### 3.4.3.3.1 Reaction Rate Constants (k) and Half‐Life (t_1_/_2_)

The control group exhibited the highest deterioration rates across all parameters, confirming rapid spoilage without antioxidants. The addition of natural or synthetic antioxidants significantly slowed deterioration, with 2CMI showing the lowest *k* values and longest t_1/2_, indicating superior stability. These findings suggest that lipid oxidation and other quality deterioration parameters are highly dependent on the presence of antioxidants. A lower *k* value indicates a slower degradation rate, while an extended t_1/2_ highlights enhanced stability over time. This trend was observed consistently across TBARS, MetMb, and microbial growth, reinforcing the protective effect of 4EAPO in delaying oxidative and microbial spoilage.

Table 10 shows a consistent decrease in *k* and increase in t_1_/_2_ when moving from Control → BHT → 1×EAPO → 4×EAPO for TBARS, MetMb and microbial counts This pattern is explained by the combined antioxidant and antimicrobial activity of EAPO: the ethyl‐acetate fraction is rich in polyphenols and oxygenated monoterpenes (e.g. β‐citronellol, geranyl derivatives) with well‐documented radical‐scavenging and membrane‐disrupting properties that both (a) directly retard lipid peroxidation and metmyoglobin formation and (b) reduce spoilage microbial activity that otherwise accelerates quality loss (Ben Akacha, Garzoli et al. 2023; Ben Hsouna et al. 2024; Ben Hsouna et al. 2023). The observed dose‐dependence in microbiological endpoints further supports this mechanism (stronger inhibition at 4× MIC). Thus, lower *k* and longer t_1/2_ at higher EAPO doses reflect slowed chemical oxidation and slower microbial proliferation.

Several studies have highlighted the role of plant extracts as natural antioxidants in meat and meat products, emphasising their ability to improve quality and extend shelf life. These extracts contribute to reducing oxidative deterioration, which aligns with the results of this study (Aziz et Karboune 2018). The kinetic modelling of antioxidant activity is a crucial aspect in evaluating the effectiveness of these natural preservatives, with the reaction rate constant (k) serving as a key parameter that reflects both the antioxidant concentration and reaction speed.

The kinetic analysis presented is empirical and has inherent limitations: only zero‐ and first‐order models were tested, the assumption of a constant rate constant *k* may not hold as antioxidant depletion or shifts in the microbial community can change reaction dynamics, and the sampling window (few time points and limited replicates) reduces the precision of the estimated *k* and *t*_1/2_. Confidence intervals for *k* were not reported, which limits formal comparison between treatments (Borowy et Ashurst 2025). For greater rigor, we recommend testing alternative formulations, increasing sampling frequency and replicates, reporting standard errors/confidence intervals for fitted parameters, and exploring coupled or multivariate models to capture oxidation microbial interactions.

#### 3.4.4 Shelf‐Life Estimation

Based on established acceptability thresholds, the estimated shelf life for each treatment was as follows: Control ∼ 9 days, BHT ∼ 14 days, 1EAPO ∼ 16 days, and 4EAPO ∼ 19 days. The control sample, which lacked antioxidant treatment, exhibited the shortest shelf life, emphasising the rapid oxidative deterioration of lipids and other quality parameters. However, incorporating BHT and EAPO with different concentrations significantly extended the shelf life, with 4EAPO demonstrating the highest protective effect. This extended stability in 4EAPO‐treated samples can be attributed to its superior antioxidant activity, which effectively delays lipid oxidation and microbial growth. These findings highlight the potential of 4EAPO as a natural alternative to synthetic antioxidants in meat preservation, ensuring prolonged freshness and quality retention throughout storage.

Similarly, a study by Konfo et al. (2023) demonstrated that extracts from plants such as cinnamon and clove extended the shelf life of chicken meat, preventing oxidative rancidity and maintaining the meat's color and texture. The present study reinforces these findings by demonstrating that 4EAPO, a plant‐derived antioxidant, offers superior protection against oxidative and microbial deterioration. Collectively, these studies underline the growing potential of plant extracts, such as EAPO, as natural alternatives to synthetic preservatives such as BHT. Plant‐based antioxidants are becoming increasingly popular in the meat industry due to consumer demand for clean products and natural preservation methods.

## 4 Conclusions

This study showed that the ethyl acetate extract of *Pelargonium odoratissimum* (EAPO) is rich in bioactive compounds, especially β‐citronellol and geranyl derivatives, which contribute to its strong antioxidant and bactericidal activities. When applied to ground beef, EAPO effectively reduced microbial growth, delayed lipid and protein oxidation, and preserved sensory qualities during cold storage. These results confirm its potential as a natural alternative to synthetic preservatives in meat products. Future research should focus on large‐scale production, safety profiling, and sensory validation of EAPO to ensure its applicability in the food industry. Comparative studies with plant‐derived preservatives, optimisation of extraction processes, and evaluation of cost efficiency will be essential for the commercial use of EAPO. Ultimately, the integration of EAPO into food preservation strategies could provide a sustainable, consumer‐friendly approach that meets the growing demand for safe, clean‐labelled food.

## Author Contributions

**Anis Ben Hsouna**: conceptualization, methodology, formal analysis, writing – original draft, writing – review and editing, supervision, data curation. **Boutheina Ben Akacha**: conceptualization, methodology, writing – original draft, writing – review and editing, supervision, data curation, visualization. **Monika Michalak**: methodology, writing – original draft, writing – review and editing. **Narjes Baazaoui**: methodology, writing – original draft, writing – review and editing. **Wirginia Kukula‐koch**: methodology, formal analysis, writing – original draft, writing – review and editing. **Wojciech Koch**: methodology, writing – original draft, writing – review and editing, formal analysis. **Rania Ben Saad**: methodology, writing – original draft, writing – review and editing, formal analysis. **Miroslava Kačániová**: methodology, writing – original draft, writing – review and editing, formal analysis, resources, project administration, funding acquisition, investigation. **Stefania Garzoli**: methodology, writing – original draft, writing – review and editing, supervision.

## Conflicts of Interest

The authors declare no conflicts of interest.

## Data Availability

The original contributions presented in the study are included in the article/supplementary material, further inquiries can be directed to the corresponding author.
